# Diverse Clinical Presentations of C3 Dominant Glomerulonephritis

**DOI:** 10.3389/fmed.2020.00293

**Published:** 2020-06-30

**Authors:** Ramy M. Hanna, Jean Hou, Huma Hasnain, Farid Arman, Umut Selamet, James Wilson, Samuel Olanrewaju, Jonathan E. Zuckerman, Marina Barsoum, Julie M. Yabu, Ira Kurtz

**Affiliations:** ^1^Division of Nephrology, Department of Medicine, UCI School of Medicine, Irvine, CA, United States; ^2^Department of Medicine, Division of Nephrology, David Geffen UCLA School of Medicine, Los Angeles, CA, United States; ^3^Department of Pathology and Laboratory Medicine, Cedars Sinai Medical Center, Los Angeles, CA, United States; ^4^Division of Renal Medicine, Department of Medicine, Brigham and Women's Hospital, Boston, MA, United States; ^5^David Geffen UCLA School of Medicine, Los Angeles, CA, United States; ^6^Department of Pathology and Laboratory Medicine, David Geffen UCLA School of Medicine, Los Angeles, CA, United States; ^7^UCLA Brain Research Institute, Los Angeles, CA, United States

**Keywords:** complement mutations, membranoproliferative glomerulonephritis, alternative pathway, C3 glomerulonephritis, proteinuria

## Abstract

C3 dominant immunofluorescence staining is present in a subset of patients with idiopathic immune complex membranoproliferative glomerulonephritis (iMPGN). It is increasingly recognized that iMPGN may be complement driven, as are cases of “typical” C3 glomerulopathy (C3G). In both iMPGN and C3G, a frequent membranoproliferative pattern of glomerular injury may indicate common pathogenic mechanisms via complement activation and endothelial cell damage. Dysregulation of the alternative complement pathway and mutations in certain regulatory factors are highly implicated in C3 glomerulopathy (which encompasses C3 glomerulonephritis, dense deposit disease, and cases of C3 dominant MPGN). We report three cases that demonstrate that an initial biopsy diagnosis of iMPGN does not exclude complement alterations similar to the ones observed in patients with a diagnosis of C3G. The first patient is a 39-year-old woman with iMPGN and C3 dominant staining, with persistently low C3 levels throughout her course. The second case is a 22-year-old woman with elevated anti-factor H antibodies and C3 dominant iMPGN findings on biopsy. The third case is a 25-year-old woman with C3 dominant iMPGN, dense deposit disease, and a crescentic glomerulonephritis on biopsy. We present the varied phenotypic variations of C3 dominant MPGN and review clinical course, complement profiles, genetic testing, treatment course, and peri-transplantation plans. Testing for complement involvement in iMPGN is important given emerging treatment options and transplant planning.

## Introduction

C3 glomerulopathy (C3G) encompasses a group of diseases that result from abnormalities in the alternative pathway of complement regulation, and has been defined by C3 only or C3-dominant immunofluorescence staining seen on renal biopsy (1). In contrast to atypical Hemolytic Uremic Syndrome (aHUS) (2), the clinical course of C3G is more indolent. As such, C3G is less likely than aHUS to present clinically as a systemically active and rapidly progressive disease (3). The term C3G was introduced to differentiate glomerular diseases which result from alternative pathway dysregulation from other immune complex mediated glomerular diseases. C3G includes C3 glomerulonephritis (C3GN) and dense deposit disease (DDD); the latter of which is characterized ultra-structurally by the presence of highly osmiophilic intramembranous deposits (4). Both C3GN and DDD often present with a membranoproliferative pattern of glomerular injury, a finding that can also be seen in thrombotic microangiopathy (TMA) (5). Also included under the “umbrella” term of C3G are a subset of cases which were historically diagnosed as immune complex mediated membranoproliferative glomerulonephritis (MPGN) of unknown etiology, but showed dominant staining for C3 by immunofluorescence staining, with lesser deposition of “typical” immune complex deposits such as IgG or IgA.

The pathogenic mechanism underlying C3G is uncontrolled production and deposition of the C3 breakdown product, C3b, along glomerular and sometimes tubular basement membranes (the latter which is most often seen in DDD) (6). While histologically the disease can appear quite heterogenous (7), pathogenically, there is a final common pathway leading to glomerular injury (8). There are important acquired forms of the disease such as autoantibodies against the regulatory proteins factor H (FH) and factor B (FB), as well as autoantibodies against the C3 convertases of the alternative and classical pathways (C3Nef and C4Nef, respectively)that can phenotypically mimic genetically acquired disorders (9–11). Patients with C5 nephritic factors (C5Nef) against downstream effectors in the final common pathway have also been reported (12). C3-5Nef and factor B antibodies have been observed in C3G patients with DDD as well as MPGN patterns on renal biopsy (9, 12–14), and can be treated with C3 and C5 blocking pharmacotherapy (15, 16).

As the understanding of the pathogenesis of C3G evolved, it became clear that some cases of immune complex mediated MPGN, including those with C3 dominant immunofluorescence staining and cases where there was also deposition of other immune complex deposits, were in fact complement-mediated, and represented a subset of C3G (1, 8, 16, 17). These cases could therefore be distinguished from cases displaying the more “typical” mixed complement and immunoglobulin deposition seen in MPGN secondary to infections and autoimmune disease, or MPGN associated with plasma cell dyscrasias and monoclonal immunoglobulin deposition disease (MIDD) (1, 10, 15, 16). In some cases, histologic features of TMA may co-exist with diagnostic features of C3G, also suggestive of abnormal complement regulation and activation as the source of glomerular disease.

Inherited or genetic causes of C3G include loss of function mutations that result in impaired self-protection from innate immunity (20), or uncontrolled activation of the alternative pathway (21–23). Mutations in Factor H, Factor I, C3, Factor B, Membrane cofactor protein (MCP), thrombomodulin (THBD), diacylglycerol kinase epsilon (DGKE) (24), and plasminogen are the more common mutations associated with DDD, C3GN, and C3 dominant iMPGN that form C3 GN (24). Table 1 summarizes the pathological findings seen in C3G and idiopathic immune complex MPGN.

**Table 1:** Pathological findings in each subtype of C3 glomerulopathy and three cases of C3 dominant idiopathic MPGN.

**Type**	**Age**	**Gender**	**Ethnicity**	**LM findings**	**IF findings**	**EM findings**	**Complement profile**	**Genetic mutations associated**	**Acquired**	**Peak UPC**	**Peak sCr**
Classic C3 GN				MPGN, TMA, MP, DEP	C3 > Ig 2:1	Varies, may have deposits	Possible increased activation	FH, FB,FI, C3,C5, THBD, PLS, MCP, DAGKE	C3/C4/C5 Nef	Inc	Inc
DDD				MPGN, TMA, Crescentic GN	C3 > Ig 2:1	Numerous dense deposits	Possible increased activation	FH, FB,FI, C3,C5, THBD, PLS, MCP, DAGKE	C3/C4/C5 Nef	Inc	Inc
C3 Dom iMPGN				MPGN, TMA	C3 > Ig 2:1	Sub End, sub Ep deposits	Possible increased activation	FH, FB,FI, C3,C5, THBD, PLS, MCP, DAGKE	C3/C4/C5 Nef	Inc	Inc
Case 1 iMPGN	41	F	Hispanic	MPGN, Endothelial swelling, Crescents	C3 > Ig	Sub End, sub Ep deposits	Low C3, normal sC5b-9	None found	None	4–6 g protein/g creatinine	3mg/dL
Case 2 C3 Dom MPGN	22	F	Asian	MPGN	C3 only	Sub End, sub Ep deposits	120–160% of normal sC5b-9	Not done	Anti CFH Ab	1–2 g protein/g creatinine	0.5 mg/dL
Case 3 C3 Dom MPGN	25	F	Hispanic	MPGN, DDD, Crescents	C3 > Ig	Dense deposits	Initially normal then decreased C3, C4	None found	None	9 g protein/g creatinine	15 mg/dl

*Anti CFH Ab, anti-complement Factor H antibody; C3G, C3 glomerulopathy; C3 Dom MPGN, C3 dominant membranoproliferative glomerulonephritis; CH50, total complement; DGKE, diacylglycerol kinase epsilon; DDD, dense deposit disease; DEP, diffuse endocapillary proliferative pattern; EM, electron microscopy; F, female; FH, factor H; FB, factor B; FI, factor I; g, gram; GN, glomerulonephritis; Inc, increased; iMPGN, idiopathic membranoproliferative glomerulonephritis; IF, immunofluorescence; Ig, immunoglobulin; iMPGN, idiopathic membranoproliferative glomerulonephritis; LM, light microscopy; M, male; MCP, membrane cofactor protein; mg/dL, milligram/deciliter; MP, mesangial proliferative pattern; MPGN, membranoproliferative glomerulonephritis [Type I]; NF, nephritic factor; PLS, plasminogen, sCr, serum creatinine [mg/dL]; Sub End, subendothelial; Sub Ep, subepithelial; SMAC, soluble membrane attack complex; THBD, thrombomodulin; TMA, thrombotic microangiopathy; UPC, urine protein/creatinine ratio [g/g creatinine]*.

We report three cases of C3G with various clinical presentations and pathologic findings. The clinical course, complement profiles, and treatment plans are reviewed. In addition, the role of eculizumab and other immunosuppressive agents is discussed as it relates to each case. The chief aim of this case series is to provide examples of the importance of detecting and managing complement dysfunction in iMPGN for the purposes of treatment and planning for renal transplantation.

## Cases

### Case 1

The patient is a 39-year-old woman who presented 1 year ago with progressive proteinuria, renal failure, and hypertension. A biopsy was performed after development of significant renal dysfunction; serum creatinine was 3 mg/dL. The protein excretion rate was 1–2 g/24 h initially, which increased to 4–6 g/24 h with worsening renal function. The initial biopsy result at an outside institution was reported as non-diagnostic, with features of chronic autoimmune glomerulonephritis. The patient had a thorough infectious disease evaluation which was negative for hepatitis A, hepatitis B virus (HBV), and hepatitis C virus (HCV) serologies, negative for human immunodeficiency virus (HIV), negative for treponemal serologies, and with no evidence of tuberculosis, coccidiomycosis or other chronic infections. She did not have known diabetes, obesity, or malignancies.

Extensive serological evaluation was negative for lupus serologies [anti-nuclear antibody negative (<1:40 titer), anti-double stranded DNA (anti dsDNA) negative (<200 international units/ml), DNAase B antibody negative (<86 Units/ml), anti-histone antibody negative 0.4 units, anti-centromere <1, ribonucleoprotein <20 units, anti-smith antibodies <20 units, Sjogren's syndrome (SSA and SSB) antibodies <20 units, rheumatoid factor negative <1, scleroderma antibodies negative at 1 AU/ml, anti-citric citrulline peptide negative at 3 units, Cardiolipin IgA, IgG, IgM was negative, and ANCA panel (c-ANCA, p-ANCA, proteinase 3 and anti-myeloperoxidase were all negative <1:20 titer). Complement titers (CH 50) were near the lower limit of normal at 43 units/mL [Reference range 42–95 Units/mL], and there was isolated depression of C3 at 67 mg/dL [Reference range 76–165 mg/dL] but with normal C4 levels at 23 mg/dL [Reference range 16–48 mg/dL]. Serum and urine electrophoretic studies and immunofixation studies did not reveal a monoclonal spike. Free light chain kappa to lambda ratio was also normal with a ratio of 1.66.

**Figure 1:**
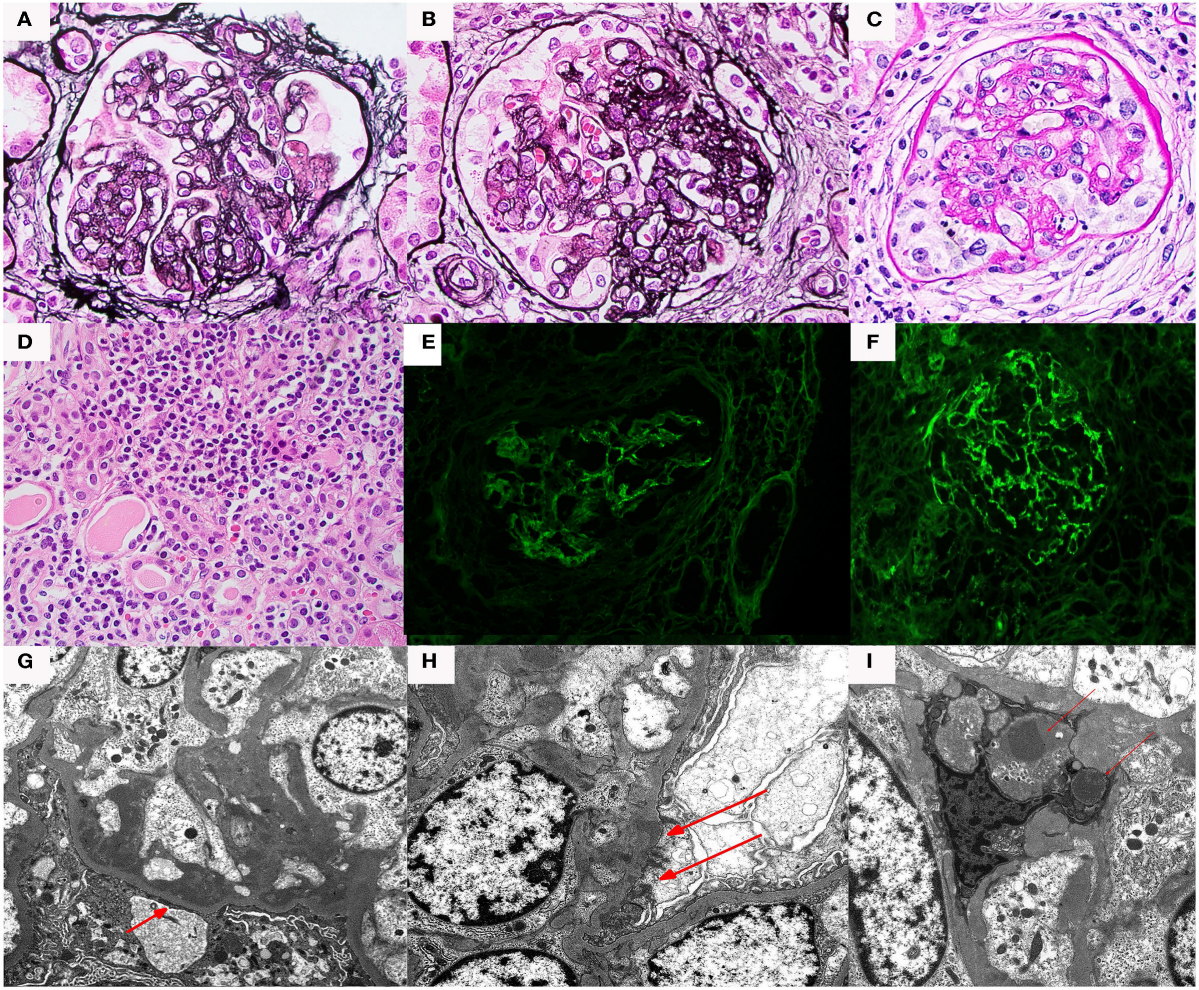
Renal biopsy data for Case 1: **(A)** light microscopy 40× silver stain Double contours and segmental scar; **(B)** light microscopy 40× silver stain Double contours with endocapillary hypercellularity and segmental scar; **(C)** light microscopy 40× hematoxylin and eosin cellular crescent with segmental karyorrhexis; **(D)** light microscopy 40× hematoxylin and eosin tubulointerstitial inflammation; **(E)** immunofluorescence IgG staining; **(F)** immunofluorescence showed C3 staining > IgG staining. **(G)** Electron microscopy, subendothelial deposits (red arrows); **(H)** electron microscopy, subepithelial deposits (red arrows); **(I)** electron microscopy, mesangial deposits (red arrows).

On renal biopsy (Figure 1), the dominant finding by light microscopy was an MPGN pattern of injury that was predominantly chronic with segmental sclerosis, extensive global glomerulosclerosis, and parenchymal scarring. However, there was residual activity including focal cellular-to-fibrocellular crescents, karyorrhexis, and diffuse, and mild endocapillary hypercellularity. Along with prominent glomerular basement membrane double contours, there was also some suggestion of mesangiolysis and marked endothelial cell swelling raising the possibility of endothelial cell injury, although definitive morphologic features of TMA were not identified. Immunofluorescence studies showed segmental to global granular staining for IgG (2+), IgA (trace), IgM (trace), C1q (2+), C3 (3+), and kappa/lambda light chains (both trace). At least two glomeruli exhibited segmental fibrinogen staining (2–3+) in areas of capillary loop necrosis. Electron microscopy revealed amorphous electron dense deposits present in a predominantly subendothelial and mesangial distribution with occasional subepithelial and intramembranous deposits. Endothelial cell cytoplasm was swollen. There were no tubuloreticular inclusions or extra-glomerular deposits. A diagnosis of immune-complex mediated membranoproliferative glomerulonephritis was rendered.

Given the negative evaluation for underlying infectious, autoimmune diseases, and neoplastic disorders, the possibility of a complement-mediated disorder was investigated. Complement profile testing did not reveal C3Nef, C4Nef, anti-factor H, or factor B antibodies. Soluble C5b-9 levels were not elevated (within the normal range of <250 ng/mL), a finding which was compatible with the patient's glomerular disease having entered a quiescent stage. No known genetic mutations or variants of uncertain significance were found at the time (Iowa complement laboratory) in the 2017 panel that was sent. The panel sent included CFH, CFI, MCP (CD46), CFB, CFHR5, THBD, C3, ADAMTS13, PLG, DGKE, MMACHC, G6PD. Multiple Ligation Probe Amplification (MLPA) was included in the testing to determine copy number variants over the complete complement Factor H related region (25).

It should be noted that the rate of capture of genetic mutations remains only 41–60%, as not all mutations leading to complement mediated disorders are known at this point (14). However, C3 levels remained low at 67–75 mg/dL a year after the renal biopsy, supporting the presence of a complement-mediated disorder. The patient has remained off renal replacement therapy and is scheduled to undergo a preemptive renal transplant. Treatment with mycophenolate mofetil with the transplant may be able to prevent any complement mediated disease process recurrence; however, further therapy would depend on demonstration of recurrent glomerulonephritis in renal allograft biopsy (26, 27).

### Case 2

**Figure 2:**
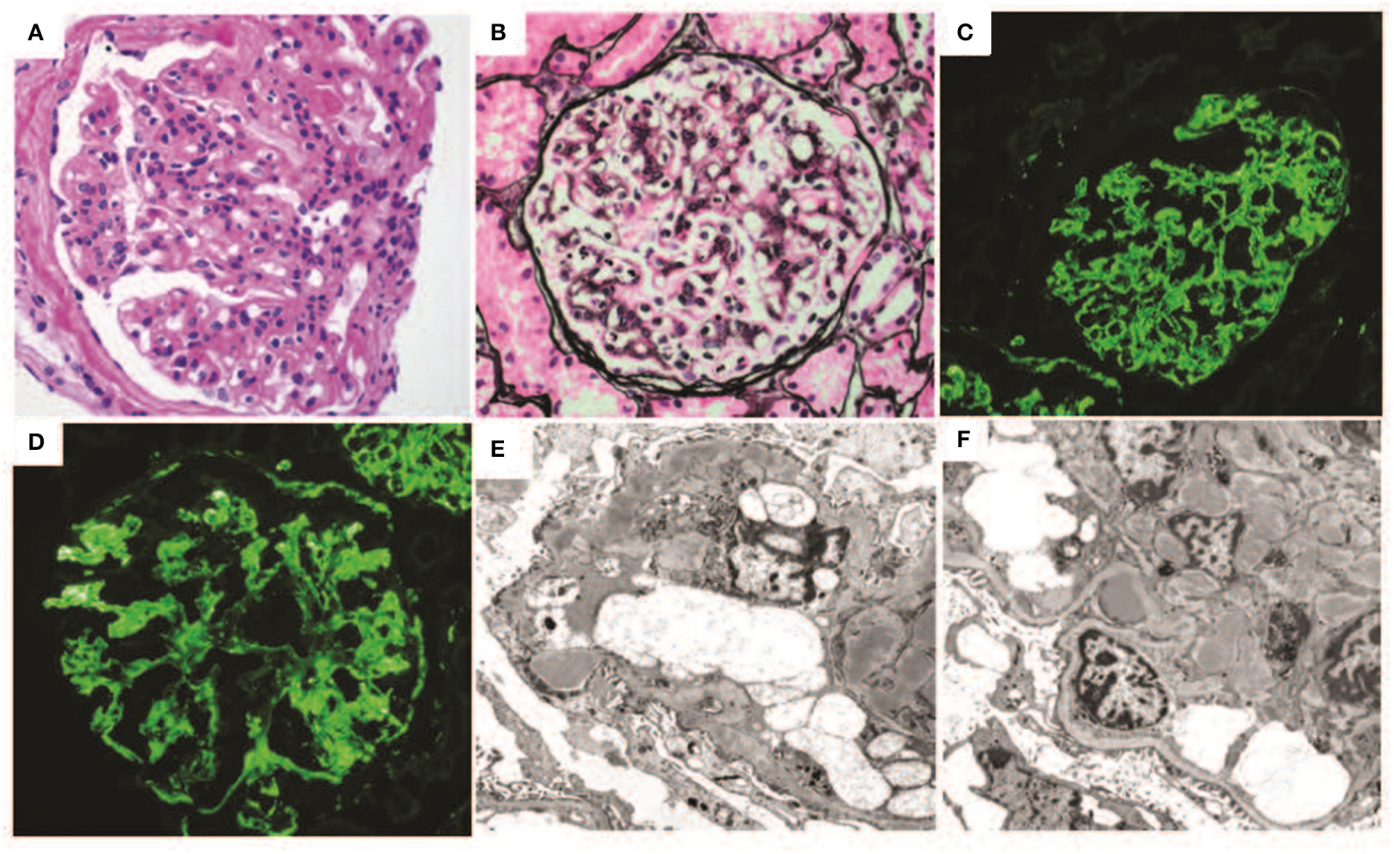
Renal biopsy data for Case 2: **(A)** light micrographs demonstrating a diffuse and global membranoproliferative glomerulonephritis pattern of injury periodic acid Schiff (PAS) stain; **(B)** Jones silver stain (40× magnification) demonstrating glomerular basement membrane double contours; **(C,D)** immunofluorescence micrographs demonstrating diffuse global bright mesangial and capillary loop C3 staining; **(E,F)** electron micrographs demonstrating numerous mesangial, subepithelial, and intramembranous deposits. Several subepithelial deposits show “hump-like” morphology. Capillary loop double contours with cellular interpositions are also present.

The patient is a 22-year-old woman who was noted to have persistent non-nephrotic proteinuria of 2–3 g protein/g creatinine with preserved renal function; serum creatinine was 0.5 mg/dL. These parameters remained essentially unchanged for 2 years, at which time a renal biopsy (Figure 2) was performed. Serum C3, C4, and CH50 were within normal range.

By conventional light microscopy, a diffuse MPGN pattern of glomerular injury was observed, with diffuse mesangial and endocapillary hypercellularity associated with conspicuous glomerular basement membrane double contour formation. Prominent mesangial and capillary loop deposits were identified. There was focal segmental glomerulosclerosis. No crescents or necrotizing features were seen. Immunofluorescence studies demonstrated bright diffuse and global capillary wall and mesangial staining for C3 without significant staining for any other immunoglobulins or complement protein. Electron microscopic evaluation demonstrated numerous amorphous electron dense deposits throughout mesangial regions, intramembranous spaces, and subepithelial spaces (including several “hump-like” deposits), and less frequently in subendothelial locations. Bowman's capsular deposits were also seen. Glomerular basement membrane duplication with mesangial interposition was also present. No highly osmiophilic intramembranous or “sausage-like” deposits were seen.

Subsequent lab tests revealed evidence for alternative pathway activation (28). Specifically, an autoantibody against complement factor H was positive at a 1:200 titer(value of _1200_AU/mL) [Reference range < 150 AU/mL] (29). An elevated soluble C5b-9 membrane attack complex (MAC) was also detected. The titer was at 1.6 (160% reference range <1). C3NeF and C4NeF were negative by immunofixation and C3/C4 degradation tests and C3 CSAP [centriole-cilia-and spindle associated protein] were also negative. Genetic testing for Cockayne syndrome B protein [CSB protein] was normal. Deletions in CFHR3-CFHR1 were screened for and not found. This testing was done since some CFHR3-CFHR1 deletions maybe associated with development of anti CFH antibodies (30). This case illustrates what is likely an acquired form of C3G.

The patient was monitored closely by her nephrologist and started on renin-angiotensin-aldosterone system (RAAS) blockade with an angiotensin receptor blocker (ARB) and an aldosterone antagonist. Anti Factor H autoantibodies remained elevated at 1:200 and was found via an ELISA assay [Reference range <150 AU/mL] (29). C5b-9 MAC levels remained elevated but improved at 1.2 (120% of reference range, <1). The patient's serum creatinine has remained normal at 0.5mg/dL, and current urinary protein-to-creatinine ratio is improved at 0.2 g protein/gram creatinine. The patient was not started on factor H inhibitor therapy or other immunosuppressive therapy given her remarkably stable renal parameters and lack of any other thrombotic manifestations. She is being monitored for deterioration at which time plasma exchange and anti CD20 monoclonal antibodies would be considered.

### Case 3

**Figure 3:**
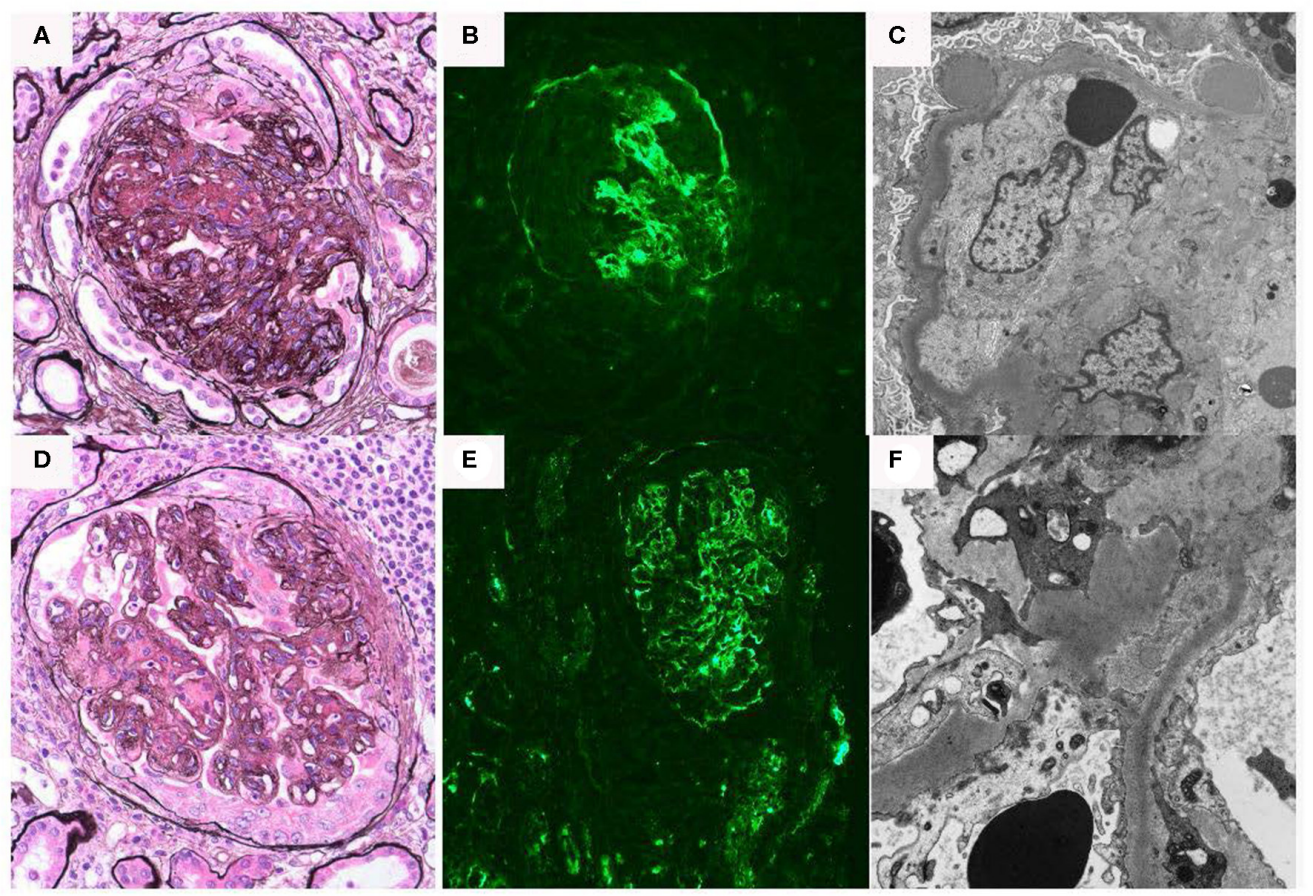
Renal biopsy data for Case 3: **(A–C)** First renal biopsy demonstrating **(A)**; light microscopy with Jones silver stain demonstrating membranoproliferative pattern of injury 40×; **(B)** immunofluorescence demonstrating dominant C3 binding in a segmental confluent granular to pseudolinear pattern; **(C)** electron microscopy showing dense deposits within segments of the basement membrane, it also shows electron dense deposit-like aggregates in scattered subepithelial and intramembranous areas; **(D–F)** second renal biopsy re-demonstrates a membranoproliferative pattern of injury, C3 dominant binding on immunofluorescence and dense deposits.

The patient is a 25-year-old woman who presented at age 16 with lower extremity edema when she was approximately six months pregnant. Kidney biopsy at that time revealed DDD with 45% active crescents and marked acute tubulointerstitial injury (Figures 3A–C). On immunofluorescence, approximately half the glomeruli had capillary wall and/or mesangial staining for C3 (4+) in a segmental confluent granular to pseudolinear pattern. On electron microscopy, capillary walls were irregular and segmentally had thickened with osmiophilic deposits which segmentally transformed and replaced the lamina densa. There were scattered aggregates of electron dense deposits in a subepithelial and intramembranous distribution, and a pathological diagnosis of DDD was made. At that time the serum creatinine was 0.99 mg/dl, with normal C3 89.5 [76–165 normal range] and C4 80.1 (16–48 normal range). The patient declined treatment at that time because of concern about taking medications during her pregnancy.

Six days after a full-term delivery, she presented to a different hospital with abdominal pain, dysuria, and worsening edema. Labs were significant for creatinine 4.1 mg/dL, albumin 1.6 g/dL, and urine protein-to-creatinine ratio of 9.3 g/g. In addition, there was isolated depression of C3 at 54.1 [76–165 normal range] with normal C4 37.5 [16–48 normal range]. A repeat kidney biopsy again showed DDD with extensive crescent formation. The tubulointerstitium showed interstitial inflammation with focal intratubular neutrophils and cellular debris suggestive of acute pyelonephritis, as well as evolving parenchymal scarring (Figures 3D–F). On electron microscopy, few and large, “hump-like” subepithelial deposits were noted. She was treated with intravenous antibiotics, one dose of intravenous methylprednisolone 500 mg, and five sessions of plasma exchange. She also started hemodialysis at that time.

Genetic testing was performed as part of a transplant evaluation. A C3 glomerulonephritis (C3GN)/DDD genetic susceptibility panel, including C3, CFB, CFH, CFHR1, CFHR2, CFHR1, CFI, MCP (CD46) identified no known mutations. In addition, C3Nef, Factors B, H, and I, Factor H autoantibody, and soluble C5b-9 levels were within normal limits (reference range <1).

The patient was activated on the kidney transplant list. After 9 years on dialysis, the patient underwent a deceased donor kidney transplant (donor in 30 s, terminal creatinine 1 mg/dl). She received basiliximab for induction immunosuppression and tacrolimus, mycophenolate mofetil, and prednisone for maintenance immunosuppression. Her post-transplant course was significant for expected delayed graft function given cold ischemia time of 19 h requiring hemodialysis for two sessions post-transplant. She was monitored closely with no evidence of proteinuria or intermittent microscopic hematuria. She is now over 1-year post-transplant with a creatinine of 0.9 mg/dl and no evidence of proteinuria or hematuria to indicate recurrent disease.

## Discussion

C3G was first described in 2007 (31, 32), and increased the awareness of the role of complement and complement dysregulation in renal disease. These diseases are continuing to be characterized in great molecular detail (17). C3G includes DDD (7, 33), C3 glomerulonephritis (34), and a subset of immune complex mediated MPGN which are thought to result from alternative pathway dysregulation. The wide variability in clinical presentation and pathology are shown in our three cases. Clinically, the presentation varied from mild to severe and progressive chronic kidney disease (CKD) requiring eventual hemodialysis. Histologically, the pathology manifestations included DDD as well as variably active and proliferative MPGN, sometimes with severe crescentic involvement. The latter finding is consistent with reports of more severe cases of C3G which have presented as crescentic and necrotizing rapidly progressive glomerulonephritis (RPGN) (27, 35), or as pulmonary renal syndromes (36). A low C3 level in iMPGN patients suggests a need for workup for alternative pathway dysfunction.

The three cases of C3G demonstrate the diversity of pathologic findings and the variability of clinical course. The first patient presented with a persistently low C3 level and had biopsy findings of MPGN and ultrastructural features suggestive of endothelial cell injury; with a more severe clinical course with significant CKD in the absence of genetic mutations in the alternative pathway. In contrast, the second patient also exhibited MPGN features on biopsy and was found to have anti-factor H autoantibodies, elevated C5b-9levels 120–160% of reference range, but with a milder clinical course not requiring immunosuppression. A limited genetic screen did not find CFHR1–3 deletions. The third patient presented with sporadic decreased C3 levels and demonstrated C3G biopsy findings with a DDD phenotype and extensive crescentic involvement. However, alternative pathway genetic testing was negative for known mutations or suspicious variants of unknown significance.

These cases highlight the etiologic, molecular, pathologic, genetic, and clinical features that demonstrate how a subset of immune complex mediated MPGN cases can overlap with diseases resulting from complement dysregulation as well as TMA (22). One important limitation of our three cases is that Pronase digestion was not used, hence, limiting ability to find “masked” immunoglobulin deposits (37). There is increasing evidence that a distinct group of C3G patients may be associated with monoclonal gammopathies, and can also manifest with histologic features consistent with C3G and MPGN (38).

Like aHUS, the genetic mutations leading to complement dysregulation in C3G are varied and interact in complex non-mendelian patterns (14, 39). There can be combinations of mutations or traits that coincide and ultimately lead to complement dysregulation. Less frequently observed are patients with mutations that are strongly heritable with a higher penetrance both in aHUS and in patients with C3G (21). It is recommended that potential organ donors be screened for complement mutations if C3G is suspected before transplant, analogous to a patient with aHUS having a family member who is a potential donor screened. This has real clinical implications as C3G has shown to frequently recur in renal allograft patients (40).

Acquired factors classically known as nephritic factors are autoantibodies that disrupt the function of various complement regulatory factors resulting in pathway dysfunction. This can ultimately lead to the uncontrolled production and deposition of complement components in the glomeruli (10, 11). The presence of nephritic factors can be found in the entire spectrum of the C3G family of disorders (10, 11, 22). In our patient group, however, we were not able to detect the presence of a nephritic factor.

It is imperative for the authors to mention that the complement testing that we did nearly 3 years ago was limited. It is now standard of genetic testing in MPGN to identify all the known genetic drivers in C3G (3). Studies have to include mutational screening of complement genes, copy number variations analyses to detect genomic rearrangements of CFHR genes, and screening of risk alleles of the CFH, MCP, and CFHR5 genes. In addition, immunological/biochemical evidence of complement involvement should be sought by measuring plasma levels of C3, C4, FH, FI, FB, and soluble C5b9 complexes, and by screening autoantibodies (anti-FH, anti-FB, C3Nef, C4Nef, C5Nef).

Treatment recommendations have been slowly evolving (26), but less targeted immunosuppression including glucocorticoids and rituximab are reportedly less effective (41, 42). There is great interest and an apparently increasing role of complement inhibition (i.e., off-label utilization of C5 convertase inhibitors such as eculizumab or other novel complement blocking agents) (43). The need to prevent recurrence post-transplant provides a compelling indication for effective treatment strategies in patients with C3G, as complement blockade has been postulated to allow an allograft to continue functioning and prevent recurrent TMA or C3G (44). Our patient with DDD who received a kidney transplant (case 3) did not require eculizumab and has not shown recurrence over 1-year post-transplant. Given the variable course of DDD, we opted not to administer eculizumab pre-transplant, but rather to clinically monitor for recurrence post-transplant. In addition, this patient remains on routine maintenance immunosuppression with mycophenolate mofetil, tacrolimus, and prednisone. It should be noted, however, that because this disease can present as a late recurrence, possibly due to an environmental trigger, vigilant screening of proteinuria and creatinine is warranted.

C3G as a persistent process related to complement dysregulation has potential analogs to aHUS beyond just diagnosis, genetics, molecular etiology, and therapy. The importance of both genetic and environmental risk factors in both diseases can explain the low penetrance and periods of quiescence seen in many cases. The finding that complement inhibition in C3G is only effective when the disease is more active might be explained by the fact that complement inhibition fails to affect quiescent or smoldering disease (44). Similarly, an environmental trigger is often needed to activate an episode or relapse of aHUS (such as pregnancy, medications, infection, multiple myeloma, and diverse rheumatological diseases) (45–47). Therefore, it is possible that C3G follows an analogous pattern where complement activating conditions can also trigger the development of C3G in genetically susceptible individuals.

## Ethics Statement

Written informed consent was obtained from the individual(s) for the publication of any potentially identifiable images or data included in this article.

## Author Contributions

RH: writing of manuscript. JH: extensive editing of manuscript. HH: report for Case 2. FA, US, JW, SO, MB, and IK: editing and writing manuscript. JZ: pathology figures and slides. JH: editing pathology figures and slides. JY: senior author. All authors contributed to the article and approved the submitted version.

## Conflict of Interest

RH is a member of the Alexion Speaker's bureau. The remaining authors declare that the research was conducted in the absence of any commercial or financial relationships that could be construed as a potential conflict of interest.

## Abbreviations

ADAMTS13: A Disintegrin and Metalloproteinase with a Thrombospondin Type 1 Motif, Member 13
aHUS: Atypical Hemolytic Uremic Syndrome
C1q: C1q Protein
C3: Complement Component 3
C4: Complement Component 4
C3G-C3: Glomerulopathy
C3GN-C3: Glomerulonephritis
CFB: Complement Factor B
CFH: Complement Factor H
CFHR1: Complement Factor H Related Protein 1
CFHR3: Complement Factor H Related Protein 3
CFHR5: Complement Factor H Related Protein 5
CFI: Complement factor I
C3Nef-C3: Nephritic Factor
C4Nef-C4: Nephritic Factor
C5Nef-C5: Nephritic Factor
C5b-9: Complement Factor 5b-9 Membrane Attack Complex
DGKE: Diacyl Glycerol Kinase Epsilon
DDD: Dense Deposit Disease
ELISA: Enzyme Linked Immunosorbent Assay
Factor B: Complement Factor B
HBV: Hepatitis B Virus
HCV: Hepatitis C Virus
IgA: Immunoglobulin A
IgG: Immunoglobulin G
IgM: Immunoglobulin M
iMPGN: diopathic, Idiopathic Immune Complex Membranoproliferative Glomerulonephritis MAC-Membrane Attack Complex
MCP: Membrane Cofactor Protein (CD-46)
MMACHC: Methylmalonic Aciduria (Cobalamin Deficiency) CblC Type, with Homocystinuria
MLPA: Multiple Ligation Probe Amplification
MPGN: Membranoproliferative Glomerulonephritis
PLG: Plasminogen
THBD: Thrombomodulin
TMA: Thrombotic Microangiopathy. Note all abbreviations formatted according to standard complement nomenclature (18, 19).
